# Pan‐cancer analyses of immunogenic cell death‐derived gene signatures: Potential biomarkers for prognosis and immunotherapy

**DOI:** 10.1002/cnr2.2073

**Published:** 2024-04-16

**Authors:** Xiaodan Han, Di Song, Yongliang Cui, Yonggang Shi, Xiaobin Gu

**Affiliations:** ^1^ Department of Radiation Oncology The First Affiliated Hospital of Zhengzhou University Zhengzhou China; ^2^ Zhengzhou University Zhengzhou China; ^3^ Department of Respiratory Medicine Zhengzhou Central Hospital Zhengzhou China

**Keywords:** immunogenic cell death, immunotherapy, pan‐cancer, prognosis, tumor microenvironment

## Abstract

**Background:**

Immunogenic cell death (ICD) is a type of regulated cell death that is capable of initiating an adaptive immune response. Induction of ICD may be a potential treatment strategy, as it has been demonstrated to activate the tumor‐specific immune response.

**Aims:**

The biomarkers of ICD and their relationships with the tumor microenvironment, clinical features, and immunotherapy response are not fully understood in a clinical context. Therefore, we conducted pan‐cancer analyses of ICD gene signatures across 33 cancer types from The Cancer Genome Atlas database.

**Methods and Results:**

We identified key genes that had strong relationships with survival and the tumor microenvironment, contributing to a better understanding of the role of ICD genes in cancer therapy. In addition, we predicted therapeutic agents that target ICD genes and explored the potential mechanisms by which gemcitabine induce ICD. Moreover, we developed an ICD score based on the ICD genes and found it to be associated with patient prognosis, clinical features, tumor microenvironment, radiotherapy access, and immunotherapy response. A high ICD score was linked to the immune‐hot phenotype, while a low ICD score was linked to the immune‐cold phenotype.

**Conclusion:**

We uncovered the potential of ICD gene signatures as comprehensive biomarkers for ICD in pan‐cancer. Our research provides novel insights into immuno‐phenotypic assessment and cancer therapeutic strategies, which could help to broaden the application of immunotherapy to benefit more patients.

## 1 INTRODUCTION

Immunogenic cell death (ICD) is a type of regulated cell death (RCD) that is capable of initiating an adaptive immune response.^1^ The currently established defining hallmark molecule of ICD is the release of damage‐associated molecular patterns (DAMPs) from dying cells. Canonical DAMPs primarily include calreticulin (CRT), high‐mobility group box 1 (HMGB1), ATP, and heat‐shock protein 70 kDa (HSP70).^2^, ^3^ The emission of DAMPs and their binding to specific pattern recognition receptors (PRRs) primes an effective T‐cell immune response during the course of ICD, involving the recruitment of antigen‐presenting cells.^4^ There is increasing evidence that treatment‐driven ICD can trigger antitumor immune responses that enhance the therapeutic effects of radiotherapy, conventional chemotherapy, and immune checkpoint inhibitor (ICI) therapy.^5^ However, the clinical utility of ICD remains less than satisfactory, which is partly attributed to the extensive use of preclinical models in the existing literature.

Single ICI therapy is not effective for up to 60% of cancer patients, and patients with specific cancers, such as melanoma, are more likely to benefit from it. Identifying ICD biomarkers to stratify patients could potentially have significant advantages, despite differences in individual immune backgrounds and various cancers. In line with this concept, numerous studies have been conducted to explore the characteristics of ICD. Accumulating evidence indicates that various DAMPs and other ICD‐related biomarkers may have prognostic and predictive value for patients affected by various tumors.^6^ In addition to the canonical DAMPs, other molecules, such as type I IFN, eIF2α, CXCL10, IL1β, IL‐17, and ANXA1, have also been considered as ICD biomarkers.^5^, ^7^, ^8^ Extensive literature reviews led to the identification of ICD‐related genes, with Abhishek et al. compiling a comprehensive summary of ICD‐derived metagene signatures that demonstrated prognostic significance in patients suffering from lung, breast, or ovarian malignancies.^9^ Building upon these metagene signatures, some studies identified ICD‐associated subtypes and validated that ICD‐related genes could predict survival and immunotherapy outcomes in head and neck squamous cell carcinoma (HNSCC), lung adenocarcinoma, glioblastoma multiforme and pancreatic cancer.^10^, ^11^, ^12^, ^13^ This collective evidence suggests that ICD‐derived metagene signatures may serve as an integrated and effective biomarker for ICD.

In our study, we incorporated these metagene signatures and established an ICD score stratification to investigate its prognostic value, clinical relevance, molecular mechanisms, tumor microenvironment associations, and response to immunotherapy across various cancers. Our research provides novel insights into pan‐cancer ICD biomarkers, which may facilitate individualized treatment decisions for physicians.

## 2 METHODS

### 2.1 Data collection

Gene expression information and clinical data for The Cancer Genome Atlas (TCGA, https://portal.gdc.cancer.gov/) pan‐cancer (33 cancer types) were downloaded from the University of California SANTA CRUZ (https://xenabrowser.net/datapages/). The abbreviations of 33 cancer names are shown in Table 1. The targeted genes of gemcitabine were obtained from the Comparative Toxicogenomics Database (https://ctdbase.org/) and Swiss Target Prediction (http://www.swisstargetprediction.ch/). The relative infiltration levels of 22 immune cells for TCGA pan‐cancer, calculated by the CIBERSORT algorithm, were downloaded from the Timer 2.0 database (http://timer.comp-genomics.org/). The microsatellite instability (MSI) status and tumor mutation burden (TMB) of pan‐cancer samples were obtained from the cBioPortal database (https://www.cbioportal.org/). The data of the immunotherapy cohort SKCM DFCI 2015 were downloaded from the cBioportal database. This dataset included patients with metastatic melanoma who had been treated with monoclonal antibodies against cytotoxic T lymphocyte‐associated antigen‐4 (CTLA4).^14^ The 34 ICD genes were obtained from a previous meta‐analysis.^9^

**TABLE 1:** Abbreviations of 33 cancer names from TCGA.

ACC	Adrenocortical carcinoma
BLCA	Bladder urothelial carcinoma
BRCA	Breast invasive carcinoma
CESC	Cervical squamous cell carcinoma and endocervical adenocarcinoma
CHOL	Cholangiocarcinoma
COAD	Colon adenocarcinoma
DLBCL	Lymphoid neoplasm diffuse large B‐cell lymphoma
ESCA	Esophageal carcinoma
GBM	Glioblastoma multiforme
HNSC	Head and neck squamous cell carcinoma
KICH	Kidney chromophobe
KIRC	Kidney renal clear cell carcinoma
KIRP	Kidney renal papillary cell carcinoma
LAML	Acute myeloid leukemia
LGG	Lower grade glioma
LIHC	Liver hepatocellular carcinoma
LUAD	Lung adenocarcinoma
LUSC	Lung squamous cell carcinoma
MESO	Mesothelioma
OV	Ovarian serous cystadenocarcinoma
PAAD	Pancreatic adenocarcinoma
PRAD	Prostate adenocarcinoma
PCPG	Pheochromocytoma and paraganglioma
READ	Rectum adenocarcinoma
SARC	Sarcoma
SKCM	Skin cutaneous melanoma
STAD	Stomach adenocarcinoma
TGCT	Testicular germ cell tumors
THCA	Thyroid carcinoma
THYM	Thymoma
UCEC	Uterine corpus endometrial carcinoma
UCS	Uterine carcinosarcoma
UVM	Uveal melanoma

### 2.2 Identification of expression differences of ICD genes

The differences in mRNA expression of the ICD gene between tumor and adjacent normal tissues were assessed for each cancer by linear regression and empirical Bayesian analysis utilizing the Limma package (Version 3.10.3) in R software. A heatmap was generated to display the logFC of gene expression values in each cancer on a *p* value threshold of <.05.

### 2.3 Correlations between ICD molecules

In the overall pan‐cancer dataset, Pearson correlation coefficients were calculated for each pair of ICD genes. Concurrently, the online STRING database (Version: 11.0, http://www.string-db.org/) was used to investigate the protein–protein interaction (PPI) network of proteins coded by ICD genes, setting a PPI score threshold of 0.4. The PPI network map was constructed with Cytoscape software (version 3.4.0, http://chianti.ucsd.edu/cytoscape-3.4.0/), and the connectivity analysis on the network nodes was performed by CytoNCA plug‐in (Version 2.1.6, http://apps. cytoscape.org/apps/cytonca).

### 2.4 Identification of transcription factors of ICD genes

The online TRRUST (version 2, https://www.grnpedia.org/trrust/) database was used to predict the potential TFs of ICD genes. The transcription factors (TFs) regulating at least five ICD genes were selected, and Sankey diagrams were created to visualize targeted relationships between these genes and their corresponding TFs.

### 2.5 Drugs targeting ICD genes

Ridge regression models were constructed to estimate the half maximal inhibitory concentration (IC50) for targeted drugs against ICD genes. This was accomplished using the pRRophetic package (version: 0.5, https://osf.io/dwzce/?action=download) in R software, which was based on the Genomics of Drug Sensitivity in Cancer (GDSC) cell line expression profiles and TCGA pan‐cancer gene expression profiles.^15^, ^16^ The Pearson correlation coefficients r were calculated between the expression level of ICD genes and the IC50 for targeted drugs. Statistical significance was defined as |*r*| > 0.3 and *p* < .05. Subsequently, the target genes of gemcitabine were predicted using the Swiss target prediction online software (http://www.swisstargetprediction.ch/) and the online Comparative Toxicogenomics Database (CTD) (https://ctdbase.org/).

### 2.6 Gene set variation analysis and gene set enrichment analysis

Fifty‐one hallmark gene sets were downloaded from the MSigDB v7.1 (http://software.broadinstitute.org/gsea/msigdb/index.jsp) database. Enrichment scores of each hallmark gene set in each sample were calculated with the gene set variation analysis (GSVA) algorithm using the GSVA package (version: 1.36.2, http://bioconductor.org/packages/release/bioc/html/GSVA.html) in R software. Subsequently, the Pearson correlation coefficients *r* between ICD gene expression and 51 hallmark pathways were calculated. Statistical significance was considered when |*r*| > 0.4 and *p* < .05. The Cytoscape (version 3.4.0) software was used for network visualization. gene set enrichment analysis (GSEA) was conducted to assess variations in the enrichment of hallmark pathways between the low and high ICD cohorts using GSEA software (http://www.broadinstitute.org/gsea/index.jsp).

### 2.7 Estimation of the immune microenvironment

The stromal score, immune score, ESTIMATE score (microenvironment score) and tumor purity was calculated using the ESTIMATE algorithm and package in R software, based on the gene expression data from TCGA pan‐cancer. Subsequently, the Spearman correlation coefficients between ICD gene expression and the three scores mentioned above were calculated for each tumor sample and visualized as a heatmap. Utilizing the relative infiltration levels of 22 immune cells, Spearman correlation coefficients were computed for each ICD gene and immune cell, with results presented in a correlation heatmap. Based on the expression levels of 47 immune checkpoints, Spearman correlation coefficients were calculated for each ICD gene and immune checkpoint gene, with the correlation heatmap displayed. Statistical significance was established at |Spearman correlation coefficient| > 0.3 and *p* < .05.

### 2.8 ICD score and correlation analysis

The ICD score, also known as the ICD gene enrichment score, was calculated for each cancer tissue and the overall TCGA pan‐cancer tissues, using the GSEA algorithm with the GSVA (version: 1.36.2) package in R software. Comparisons of ICD scores between tumor samples and normal samples were conducted on the overall TCGA pan‐cancer samples and individual tumor sample through the t‐tests. Based on the TMB and MSI data of TCGA pan‐cancer samples, Spearman correlation coefficients between the ICD score and the TMB or MSI were calculated for each tumor sample. Based on the expression levels of 47 immune checkpoint genes, Pearson correlation coefficients between the ICD score and immune checkpoint genes were calculated for each tumor sample, resulting in a correlation heatmap. The high ICD cohorts had ICD scores above the median, while the low ICD cohorts had ICD scores below the median.

### 2.9 Functional enrichment analysis

Differentially expressed genes (DEGs) were identified based on adjusted *p* < .05 and |fold change| > 2. Subsequently, Gene Ontology (GO) and Kyoto Encyclopedia of Genes and Genomes (KEGG) analyses were performed to compare the differential biological effects and signal pathways using the Metascape database (https://metascape.org/gp/index.html), Database for Annotation, Visualization and Integrated Discovery (https://david.ncifcrf.Gov), and the OmicsBean database (www.omicsbean.cn/). The enrichment analyses were conducted with a *p* value threshold of <.05.

### 2.10 Survival analysis

Kaplan–Meier analysis was conducted to compare the overall survival (OS) between the low ICD and high ICD cohorts, utilizing the survival packages (Version 2.41.1) in R software. Each cancer sample was divided into a high ICD cohort and a low ICD cohort based on the median ICD score. Univariate and multivariate Cox regression analyses were conducted, and variables with *p* < .05 in multivariate Cox regression analysis were considered as independent risk factors. Based on the independent risk factors, an OS predictive nomogram and calibration curves were generated using the Rms package in R software. *p* < .05 was considered statistically significant.

### 2.11 Statistical analysis

A t‐test was conducted to compare the differences in ICD scores between cancer patients receiving radiation therapy and those not receiving it. The Wilcoxon test was employed to compare the differences in the stromal score, immune score, ESTIMATE score, and tumor purity between the low and high ICD cohorts. For categorized variables, differences between the two groups were compared using the Wilcoxon test, while those among multiple groups were compared using the Kruskal–Wallis test. *p* < .05 was considered statistically significant.

## 3 RESULTS

### 3.1 Identification of differentially expressed ICD genes and prognostic analysis in pan‐cancer

**FIGURE 1:**
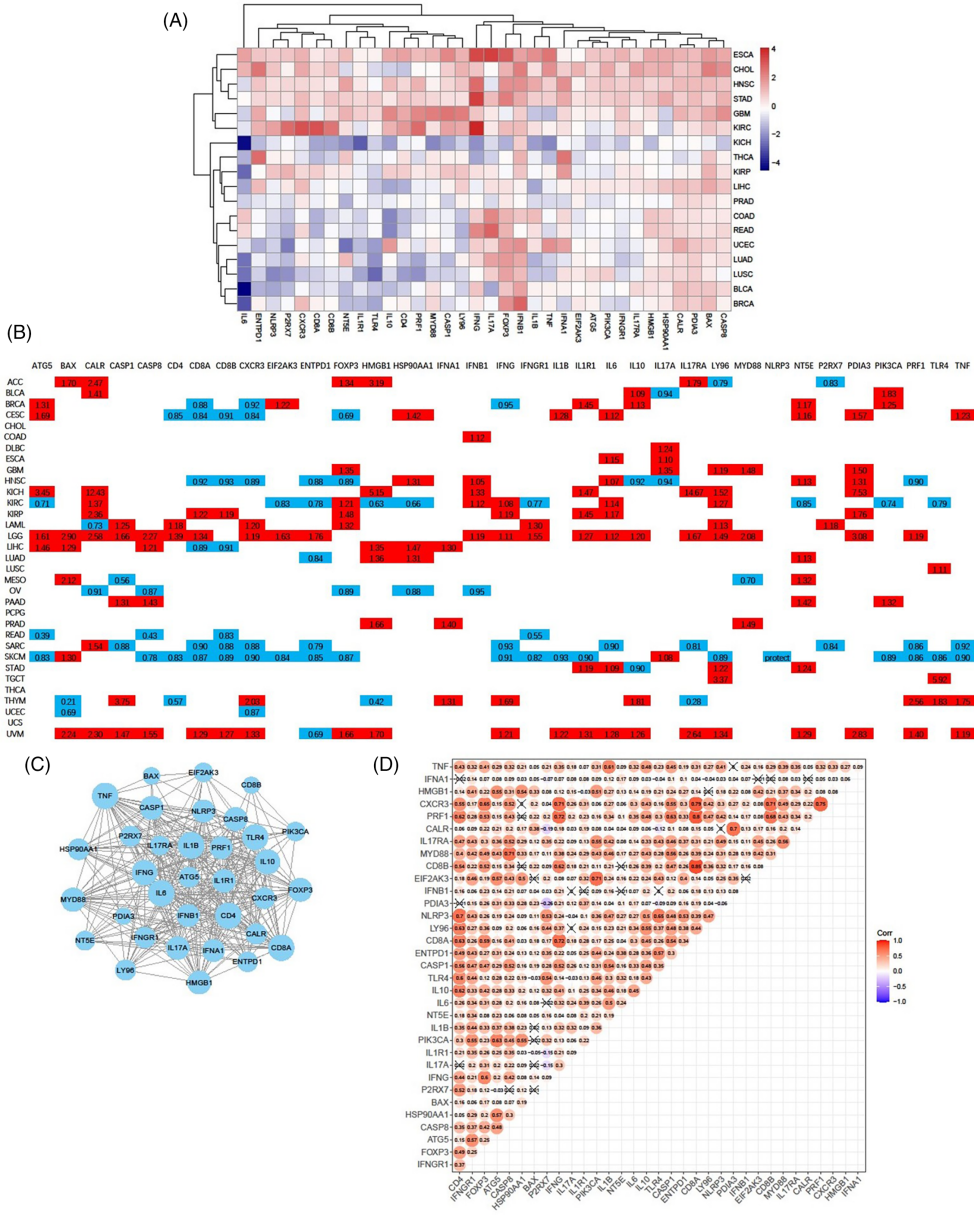
Identification of differentially expressed immunogenic cell death (ICD) genes and prognostic analysis in pan‐cancer. (A) Heatmap showing the expression differences of the ICD genes between primary tumor and adjacent normal tissues, which was based on the log2 (fold change) of 18 cancer types that had more than five normal samples. (B) Diagram of univariate Cox regression analysis showing the role of ICD genes in survival, either as a risk (hazard risk [HR] > 1) or protective (HR <1) factor. (C) PPI networks among the proteins encoded by ICD genes. (D) Correlations among the expressions of ICD genes. The colors of scatter dots range from blue to red, indicating a correlation coefficient (the number on the scatter dots) ranging from negative to positive. The cross on scatter dots indicates insignificance. The Pearson's Correlation test was used for *p* value calculation. Statistical significance was set at *p* < .05.

The differential mRNA expression levels of 34 ICD genes were evaluated between tumor and adjacent normal tissues, based on a previous meta‐analysis.^9^ The analysis was conducted across cancer types with more than five normal samples. Eighteen cancer types were included in the analysis, and our findings revealed that the majority of the 34 ICD genes were up‐regulated in ESCA, CHOL, HNSC, STAD, GBM, and KIRC (Figure 1A). In contrast, numerous ICD genes were down‐regulated in some other cancers, especially in KICH. At the genetic level, *IFNB1*, *IFNG*, *IFNA1*, *CXCR3*, *FOXP3*, *CASP8*, *PDIA3*, and *BAX* were up‐regulated in most cancers. It is well established that type I interferons, *IFNB1* and *IFNA1*, play a crucial role in the anti‐tumor immune response.^17^ The expression of *IFNB1*, as shown in Figure S1A, was significantly elevated in 10 cancers. *IL6*, an oncogenic cytokine, was notably down‐regulated in 10 cancers (Figure S1B).

We further evaluated the prognostic significance of ICD genes in 33 cancers derived from the TCGA database. The majority of ICD genes exhibited comparable association with OS, and more than half of the 34 ICD genes were found to be correlated with poorer survival for patients with LGG and UVM (Figures 1B and S1C). Eighteen and eleven ICD genes were associated with better survival for patients with SKCM and SARC, respectively. Notably, *CXCR3*, *CD8B*, and *ENTPD1* were identified as potent protective factors against six cancers. In contrast, *IL6*, *CLAR*, *LY96*, and *NT5E*, were determined to be significant risk factors for eight cancers. Our findings underscore the expression discrepancies and diverse prognostic implications of genes involved in ICD gene signatures, indicating their pivotal roles across a spectrum of cancers.

### 3.2 Identification of correlations between ICD genes and prediction of TFs

To further elucidate the correlations between ICD genes, PPI networks and correlation plots were generated to visualize these associations. Protein–protein interactions were prevalent among the 34 ICD gene‐coding proteins; particularly IL1B, TNF, IL6, CD8A, and CD4 (Figure 1C). We observed strong positive correlations between the majority of ICD genes (Figure 1D). Among the significantly relevant genes, *CXCR3* displayed close associations with *IFNG*, *CD8A*, *CD8B*, *PRF1*, and *FOXP3* (Figure 1D and Figure S2A). Additionally, *PRF1* also exhibited high correlations with *CXCR3*, *IFNG*, *CD8A*, and *CD8B* (Figure S2B).

To investigate the transcriptional regulation of ICD genes, we predicted direct upstream TFs. A Sankey diagram was constructed to display the correlation between ICD genes and their upstream TFs, reflecting the transcriptional regulation of more than five ICD genes (Figure S2C). Notable TFs such as CREB1, IRF1, JUN, NFKB1, RELA, SP1, and STAT1 may play significant roles in the transcriptional regulation of ICD genes, including *IFNG*, *IL6*, *IL10*, *FOXP3*, *IFNA1*, and *IFNB1*.

### 3.3 Pathway enrichment analysis and prediction of therapeutic agents targeting ICD genes

**FIGURE 2:**
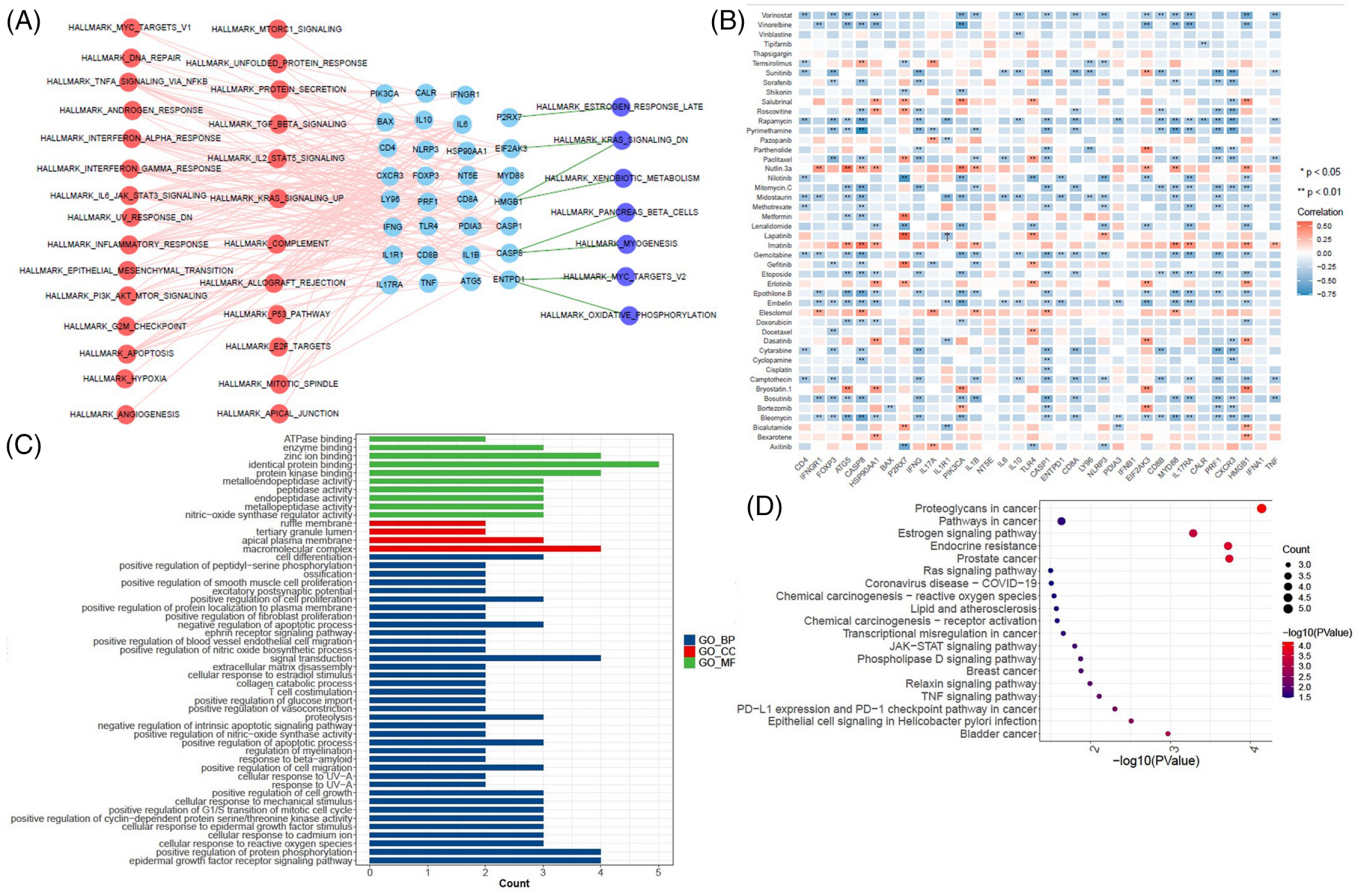
Pathway enrichment analysis and prediction of therapeutic agents targeting immunogenic cell death (ICD) genes. (A) Correlations between the ICD genes and 51 cancer hallmark pathways. Red represents hallmark pathways positively correlated with ICD gene expression, while purple represents hallmark pathways negatively correlated with ICD gene expression. (B) Correlations between 47 chemotherapeutic agent activities and the expression of the ICD genes. The Pearson's Correlation test was used for p‐value calculation. (C) Gene Ontology enrichment analysis of 11 targeted genes, including biological process (BP, blue bars), cell component (CC, red bars) and molecular function (MF, green bars) categories. (D) Kyoto Encyclopedia of Genes and Genomes enrichment analysis of 11 targeted genes. Statistical significance was set at *p* < .05.

We further examined the relationship between 51 cancer hallmark‐related pathways and ICD genes to enhance our understanding of the impact of ICD genes on cancer progression. The majority of the 51 hallmark pathways were positively correlated with ICD genes. The results showed that *CD8A*, *LY96*, *PRF1*, and *CXCR3* had strong positive relationships with the inflammation response, IL2/STAT5 signaling pathway, IL6/JAK/STAT3 signaling pathway, and complement pathway (Figure 2A). Notably, *IFNG* and *PRF1* both showed significant positive correlations with the interferon alpha response and interferon gamma response, in addition to the IL6/JAK/STAT3 signaling pathway. *CD8A* was also positively associated with the interferon alpha response, and *CD8B* was positively related to the interferon gamma response. These findings confirm the close connection between ICD genes and cancer signaling pathways.

To predict therapeutic agents targeting ICD genes, we analyzed the IC50 values of 47 common chemotherapeutic agents in each sample to indicate sensitivity to these drugs. Among the 47 chemotherapeutic agents, the IC50 values of gemcitabine, bleomycin, rapamycin, embelin, and vorinostat, were negatively correlated with the expression of more than 15 ICD genes (Figure 2B). These results suggest that cancers, such as ESCA, CHOL, HNSC, STAD, GBM, and KIRC, with high expression of ICD genes, may be more sensitive to these drugs. The IC50 values of nutlin.3a and imatinib were positively correlated with the expression of more than eight ICD genes (Figure 2B). These results suggest that ESCA, CHOL, HNSC, STAD, GBM, and KIRC, with high expression of these ICD genes, may be resistant to nutlin.3a and imatinib.

Gemcitabine, a deoxypyrimidine analogue, exhibits antitumor effects by inhibiting DNA synthesis and has been widely used as a chemotherapeutic agent. It has been proposed to increase the immunogenicity of tumor cells and modulate the tumor microenvironment.^18^, ^19^, ^20^ Therefore, we analyzed the possible target genes of gemcitabine using the Comparative Toxicogenomics Database and Swiss Target Prediction. GO and KEGG enrichment analyses were performed on the 11 target genes (*SLC29A1*, *EGFR*, *CDA*, *AKT1*, *TYMP*, *MMP9*, *ADAM17*, *ESR1*, *IGFBP3*, *MMP3*, and *PTPN11*) sourced from both databases (Figure 2C,D). These genes were mostly enriched in biological processes, particularly in the PD‐L1 expression and PD1 checkpoint pathway in cancer, TNF signaling pathway, JAK–STAT signaling pathway, and epidermal growth factor receptor signaling pathway. These results suggest that these therapeutic agents targeting ICD genes, especially gemcitabine, may be regarded as ICD inducers.

### 3.4 Evaluation of correlations between ICD genes and tumor microenvironment

**FIGURE 3:**
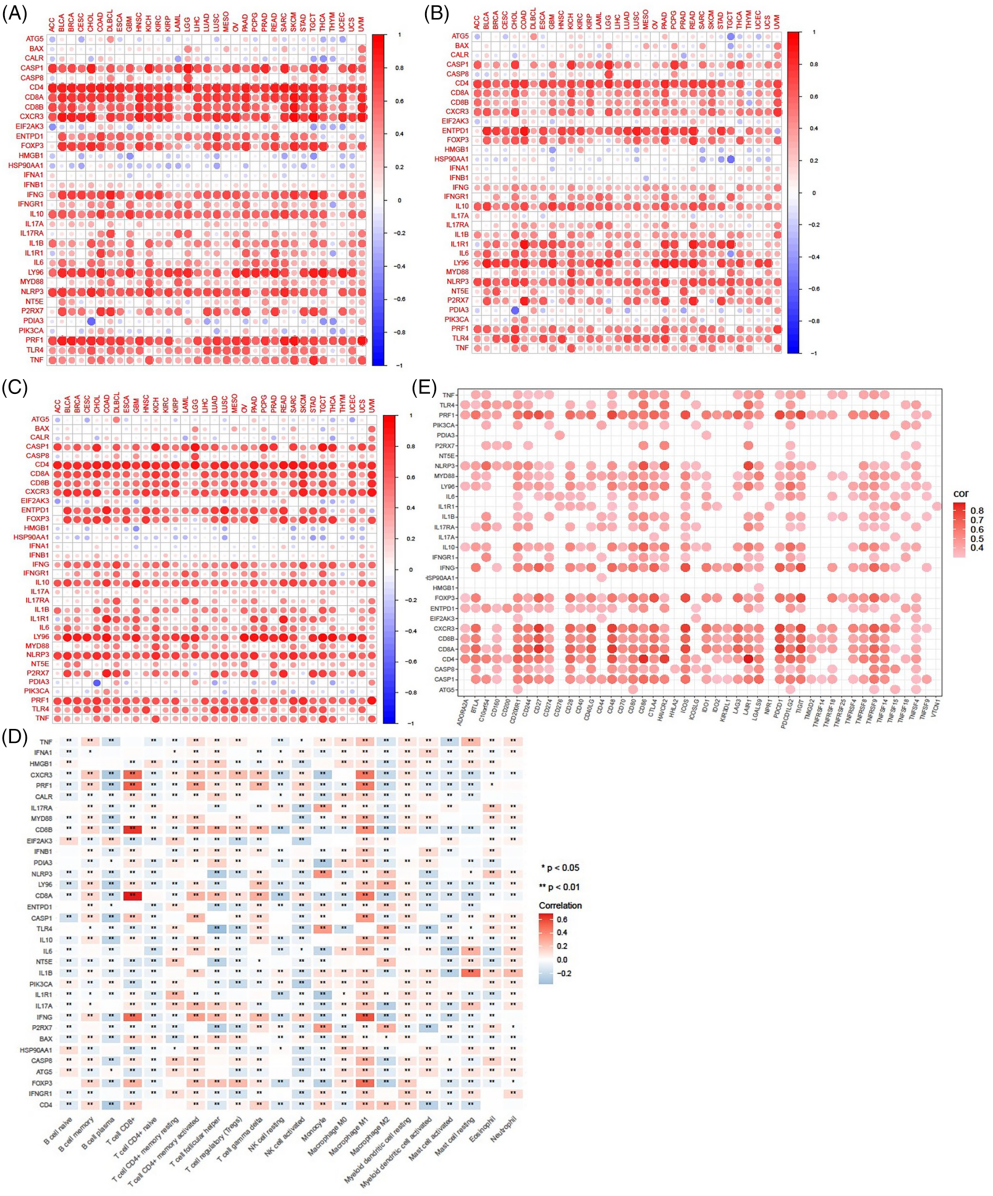
Correlations between immunogenic cell death (ICD) genes and the tumor microenvironment. (A) Correlations between ICD genes and the immune scores. (B) Correlations between ICD genes and stromal scores. (C) Correlations between ICD genes and ESTIMATE scores. (D) Correlations between ICD genes and infiltration levels of 22 immune cells. (E) Correlations between ICD genes and 47 immune checkpoints. The Spearman correlation test was used for *p* value calculation. Statistical significance was set at *p* < .05.

In the tumor microenvironment, immune cells and stromal cells, representing two major nontumor components, have been suggested as valuable references for tumor diagnosis and prognostic prediction.^21^ To validate the association between ICD genes and the tumor microenvironment, we calculated the immune scores and stromal scores to estimate the proportion of infiltrating immune and stromal cells in 33 cancer tissues. The ESTIMATE scores were then obtained by combining of immune scores and stromal scores. As shown in Figure 3A–C, the expression levels of *CD4*, *CD8A*, *CD8B*, *CXCR3*, *FOXP3*, *IFNG*, *LY96*, *NLRP3*, and *PRF1* were highly correlated with the immune scores, stromal scores, and ESTIMATE scores in the vast majority of 33 cancers. We further evaluated the relationships between ICD genes and the infiltration levels of 22 immune cells in 33 cancer tissues. As shown in Figure 3D, M1 macrophages and CD4^+^ memory activated T cells showed significant positive correlations with most ICD genes, while M2 macrophages, plasma B cells, and CD4^+^ naïve T cells exhibited significant negative correlations with several ICD genes. The important genes highly associated with CD8^+^ T cells, which are critical effector cells in antitumor immune response, included *CD8A*, *CD8B*, *CXCR3*, *PRF1*, *IFNG*, *FOXP3*, *CASP1*, and *CD4*. Interestingly, the expression profiling of these eight genes also demonstrated significant positive correlations with M1 macrophage infiltration. In addition, we demonstrated the correlation between ICD genes and common immune checkpoints. As shown in Figure 3E, there were significant positive relationships between most ICD genes and immune checkpoint genes. The expression levels of *CD4*, *CD8A*, *CD8B*, *CXCR3*, *PRF1*, and *FOXP3* were strongly positively correlated with several immune checkpoint genes, such as *PDCD1*, *TIGIT*, *CD200R1*, *CD27*, *CD48*, and *ICOS*. These results suggest that ICD genes, especially *CD4*, *CD8A*, *CD8B*, *CXCR3*, *PRF1*, and *FOXP3*, may be largely involved in the regulation of the tumor microenvironment.

### 3.5 Identification of the prognostic significance and clinical relevance of ICD scores

**FIGURE 4:**
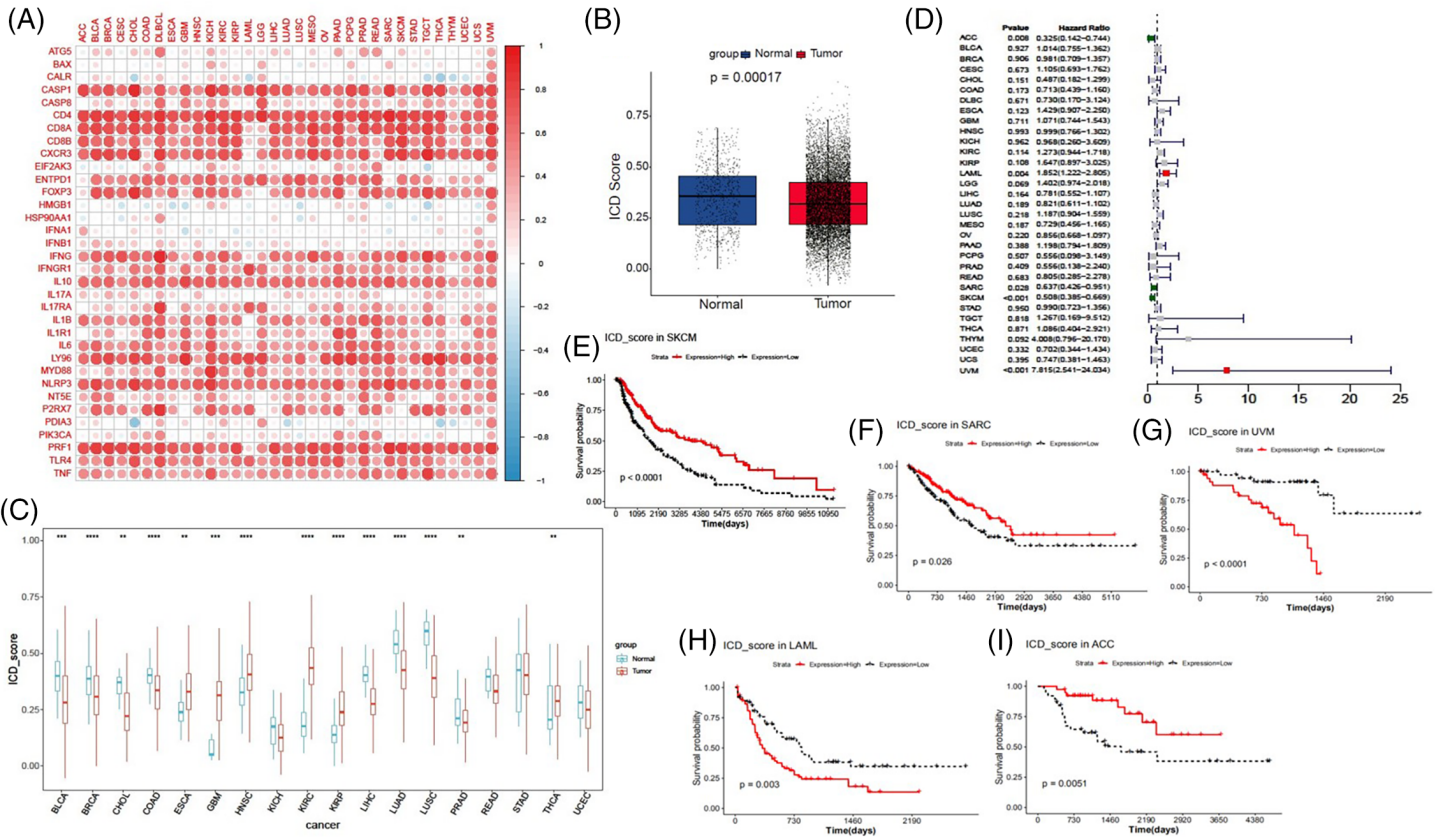
Prognostic significance of immunogenic cell death (ICD) scores. (A) Correlations between ICD scores and ICD gene expression. ICD scores of tumor samples and normal samples in the overall pan‐cancer dataset (B), as well as different cancer types (C), were compared using the *t* test. (D) Univariate Cox analysis evaluating the prognostic significance of ICD scores in terms of overall survival. Forest plots showing the hazard risk with 95% confidence intervals in OS. (E–I) Kaplan–Meier OS curves of ICD scores in the indicated cancer types. Statistical significance was set at *p* < .05.

To determine whether ICD genes contribute to clinical risk prediction, we first computed the ICD score for each cancer type and the overall TCGA pan‐cancer. To verify whether the ICD score could predict the ICD state in different cancer types or pan‐cancer, we examined the relationships between ICD scores and ICD gene expression. As showed in Figure 4A, the majority of ICD genes exhibited a strong positive correlation with the ICD score of different cancer types, particularly *CD4*, *IFNG*, *CASP1*, *CD8A*, *LY96*, *CD8B*, *PRF1*, and *CXCR3*. In contrast, a few individual genes, such as *BAX*, *CLAR*, *HMGB1*, *HSP90A11*, *IFNA1*, and *PDIA3*, showed weak correlations with the ICD scores in most cancer types. We further compared the ICD scores of tumor samples and normal samples in the overall pan‐cancer dataset and across different cancer types. The overall ICD score was consistently down‐regulated in tumor tissues compared to the adjacent normal tissues (Figure 4B). The ICD scores of BLCA, BRCA, CHOL, COAD, LIHC, LUAD, and LUSC were also down‐regulated in tumor tissues compared to normal tissues. However, the ICD scores of ESCA, GBM, HNSC, KIRC, KIRP, and THCA were up‐regulated in tumor tissues compared to normal tissues (Figure 4C). These findings imply that the ICD state varies among different cancer types, and that some individual cancers exhibit a high ICD state in the context of a low ICD state across pan‐cancer.

To further elucidate the prognostic value of ICD scores, we performed survival analysis and univariate Cox regression analysis in TCGA 33 cancer types. We found that ICD scores served as a potent protective factor for ACC, SARC, and SKCM, but acted as a risk factor for LAML and UVM (Figure 4D). The survival curves for these five cancers are presented in Figure 4E–I.

**FIGURE 5:**
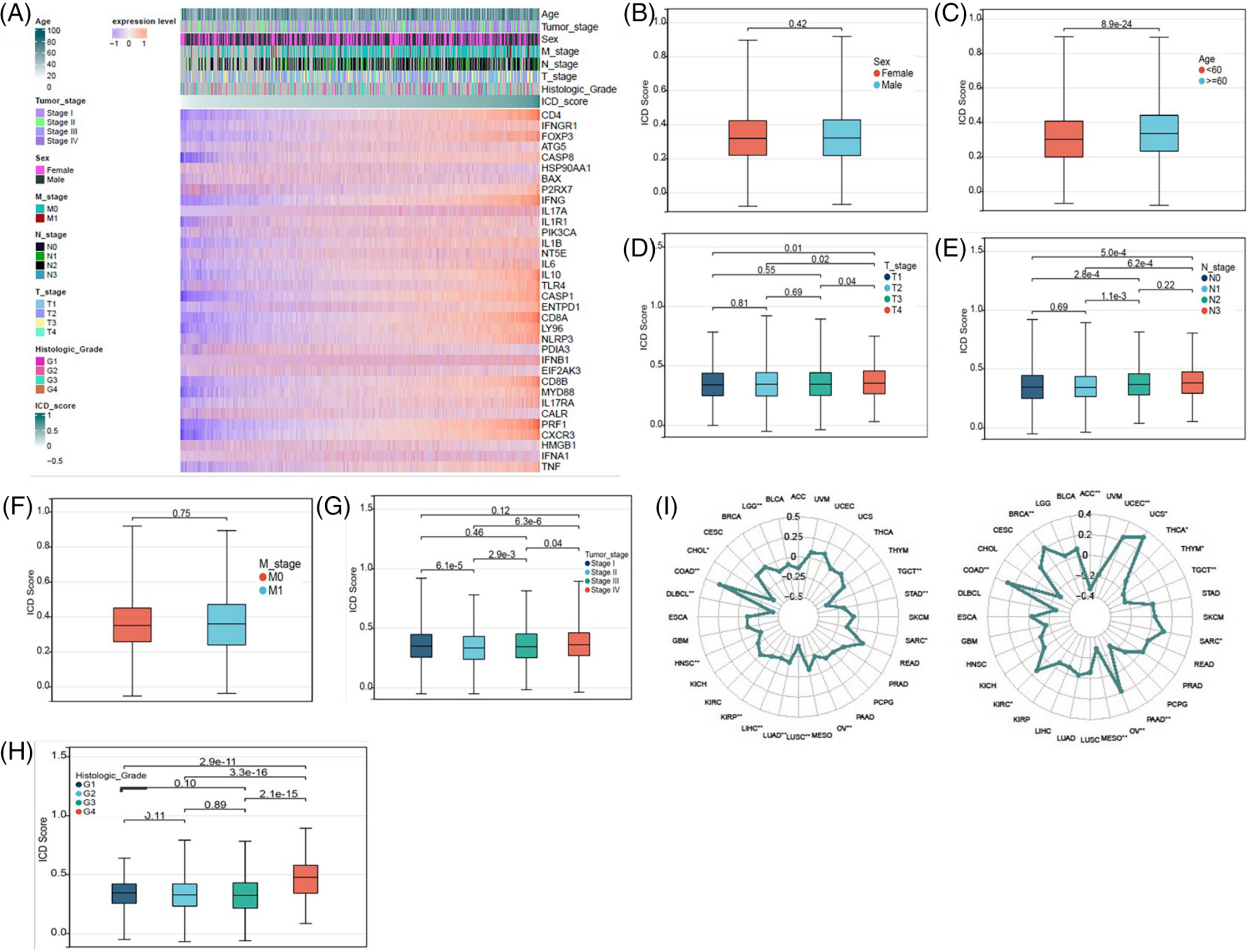
Clinical relevance of immunogenic cell death (ICD) scores. (A) Pan‐cancer heatmap showing patients' clinical features based on the ICD score. The ICD scores of patients with different sexes (B), ages (C), T stages (D), N stages (E), M stages (F), clinical stages (G), and histological grades (H) in pan‐cancer. The differences in sex, age and M stages were compared using the Wilcoxon test, whereas the differences in T stages, N stages, clinical stages and histological grades were compared using the Kruskal‐Wallis test. (I) Correlations between ICD scores and MSI (left) and TMB (right) in pan‐cancer. The Spearman correlation test was used for *p* value calculation. Statistical significance was set at *p* < .05.

To assess the clinical significance of ICD scores, we compared the ICD scores in different cohorts according to the clinical information of cancer patients, including age, sex, TNM stage, clinical stage, and histologic grade (Figure 5A). As shown in Figure 5B,C, patients under 60 years of old had higher scores than those older than 60 years old, while there was no difference in ICD scores between male and female patients. Patients with T4 stage had higher ICD scores than those with T1–T3 stages, although no significant differences were observed between patients with T1–T3 stages (Figure 5D). Patients with N2–N3 stages had higher ICD scores than those with N0–N1 stages, yet no noticeable difference was seen between patients with M0 and M1 stages (Figure 5E,F). In clinical stages II‐IV, ICD scores increased with the clinical stage. Interestingly, patients with clinical stage I had higher ICD scores than those with stage II, and no significant differences were found between patients with stage II and those with stage III or IV (Figure 5G). Patients with histologic grade G4 had higher ICD scores than those with grades G1–G3, but no significant differences were demonstrated between patients with histologic grades G1–G3 (Figure 5H). We also evaluated the relevance of ICD scores to the MSI or TMB. We found that the MSI was positively associated with ICD scores in COAD, while it was negatively correlated with ICD scores in CHOL, DLBCL, LUSC, and TGCT (Figure 5I). The TMB positively correlated with ICD scores in COAD, UCS, UCEC, and BRCA, while negative correlations were observed between the TMB and ICD scores in ACC, MESO, PAAD, and TGCT (Figure 5I). These results indicate that ICD scores have important clinical implications.

### 3.6 Evaluation of correlations between ICD scores and tumor microenvironment

**FIGURE 6:**
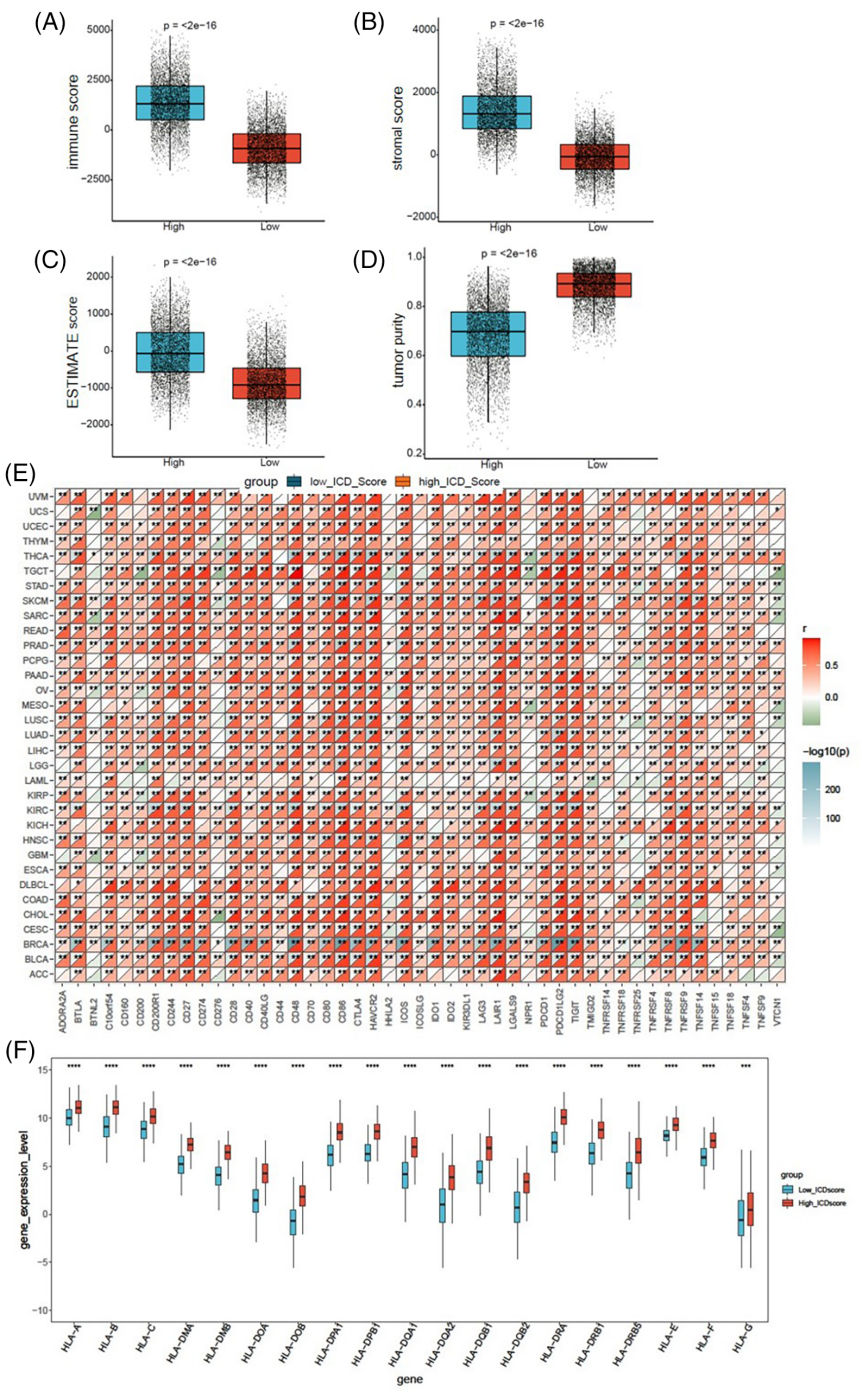
Correlations between immunogenic cell death (ICD) scores and the tumor microenvironment. The immune score (A), stromal score (B), ESTIMATE score (C), and tumor purity (D) in high ICD cohorts versus low ICD cohorts in pan‐cancer were compared using the Wilcoxon test. (E) Correlations between ICD scores and 47 immune checkpoints across 33 cancer tissues. The Pearson's Correlation test was used for p‐value calculation. (F) The expression of and HLA genes in high ICD cohorts versus low ICD cohorts in SKCM samples from TCGA was compared using the Wilcoxon test. Statistical significance was set at *p* < .05.

To elucidate the relevance of ICD scores within the tumor microenvironment, we conducted a comparative analysis of the immune score, stromal score, ESTIMATE score, and tumor purity between low and high ICD cohorts in the overall TCGA pan‐cancer samples. The high ICD cohorts exhibited significantly higher immune scores, stromal scores, and ESTIMATE scores (Figure 6A–C), while the tumor purity was lower compared to the low ICD cohorts (Figure 6D). Concurrently, we assessed the correlation between ICD scores and 47 immune checkpoints across 33 cancer tissues. The ICD scores of BRCA, SKCM, UVM, and THCA demonstrated strong positive correlations with over 23 immune checkpoints, with the correlation coefficients greater than 0.6. There were close relationships between ICD scores and CD48, CD86, CTLA4, HAVCR2, ICOS, LAIR1, PDCD1LG2, TIGIT, and TNFRSF9 in the majority of cancers (Figure 6E). We next examined the infiltration levels of 22 kinds of immune cells in SARC patients from TCGA. SARC patients in high ICD cohorts exhibited significantly elevated percentages of CD8^+^ T cells, gamma delta T cells, memory activated CD4^+^ T cells, M1 macrophages, M2 macrophages, and neutrophil cells (Figure S3A). Particularly, the ratios of M1 and M2 macrophage percentages were higher in high ICD cohorts compared to low ICD cohorts in SARC (Figure S3B). In addition, the human leukocyte antigen (HLA) genes were significantly up‐regulated in high ICD cohorts in SKCM samples from TCGA (Figure 6F). HLA genes play an important role in regulating immune infiltration and are associated with tumor prognosis and immunotherapy response.^22^, ^23^ These data imply that the ICD score may serve as a potential marker for tumor microenvironment.

### 3.7 Determination of the relationship between ICD scores and radiotherapy

**FIGURE 7:**
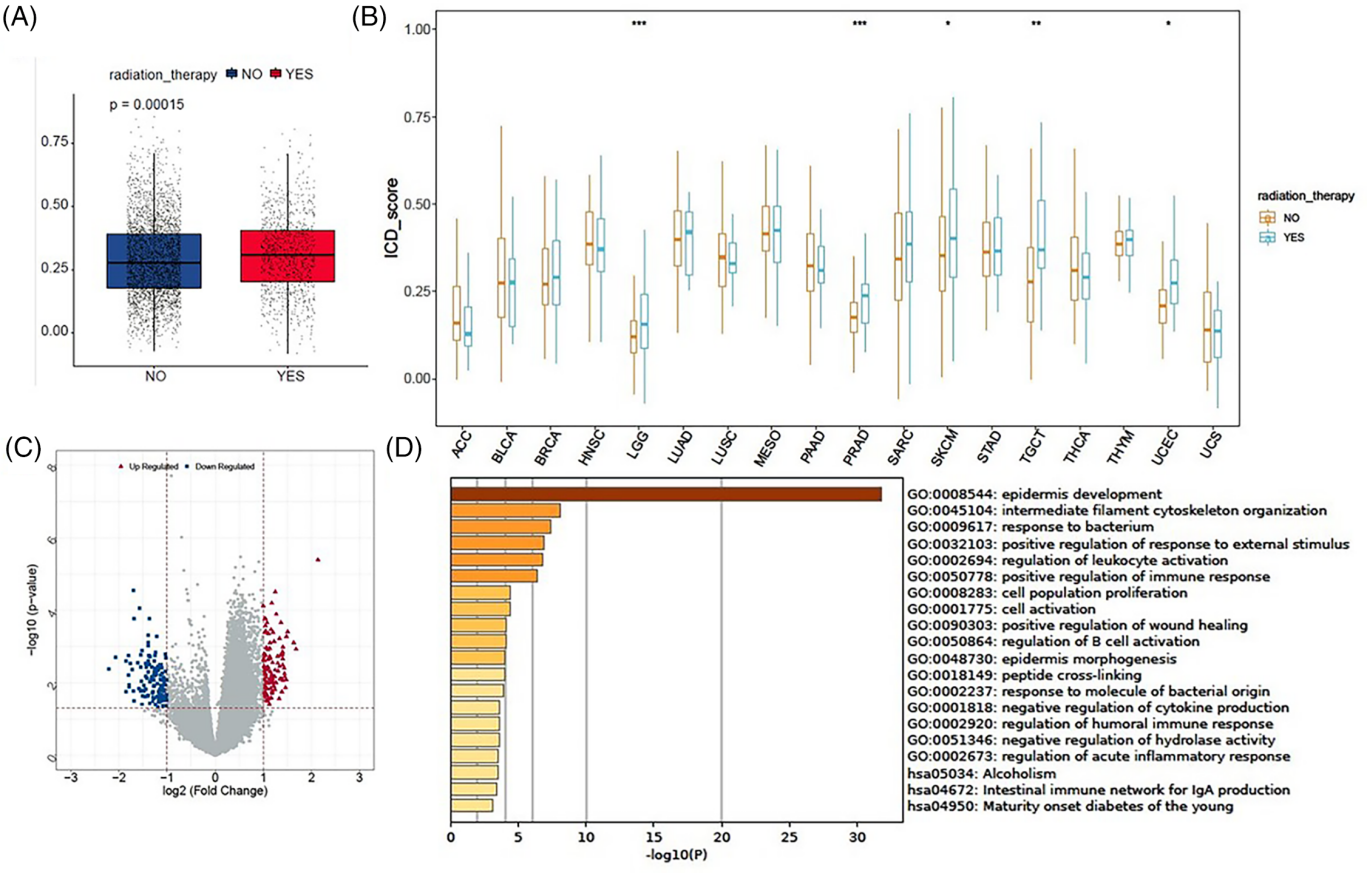
Correlations between immunogenic cell death (ICD) scores and radiotherapy. ICD scores of patients who received radiotherapy versus those who did not in the overall pan‐cancer cohort (A), as well as indicated cancer types where radiotherapy data is available (B), were compared using the *t* test. (C) A volcano plot presenting the distribution of DEGs between patients who received radiotherapy and those did not. (D) Gene ontology and Kyoto Encyclopedia of Genes and Genomes enrichment analyses of differentially expressed genes. Statistical significance was set at *p* < .05.

The abscopal effect of radiotherapy has been reported to be associated with a radiation‐driven antitumor immune response. We assessed the relationship between radiotherapy and ICD scores to explore the significance of radiotherapy in inducing ICD. The ICD scores of patients receiving radiotherapy were observed to be higher than those of patients not receiving radiotherapy in the overall TCGA pan‐cancer samples (Figure 7A). Similarly, the ICD scores were elevated in patients undergoing radiotherapy compared to those not receiving radiotherapy in LGG, PRAD, SKCM, TGCT, and UCEC (Figure 7B). As shown in Figure 7C,D, we identified the DEGs in SKCM and conducted GO and KEGG analyses. These DEGs were primarily enriched in immune‐related biological processes, including the regulation of leukocyte activation, positive regulation of immune response, negative regulation of cytokine production, regulation of humoral immune response, regulation of acute inflammatory response, and intestinal immune network for IgA production. In summary, our findings suggest that radiation serves as an ICD inducer and that the radiation‐driven antitumor immune response might be associated with inducing ICD.

### 3.8 The immunogenic features between the high ICD cohorts and low ICD cohorts in an immunotherapy cohort

**FIGURE 8:**
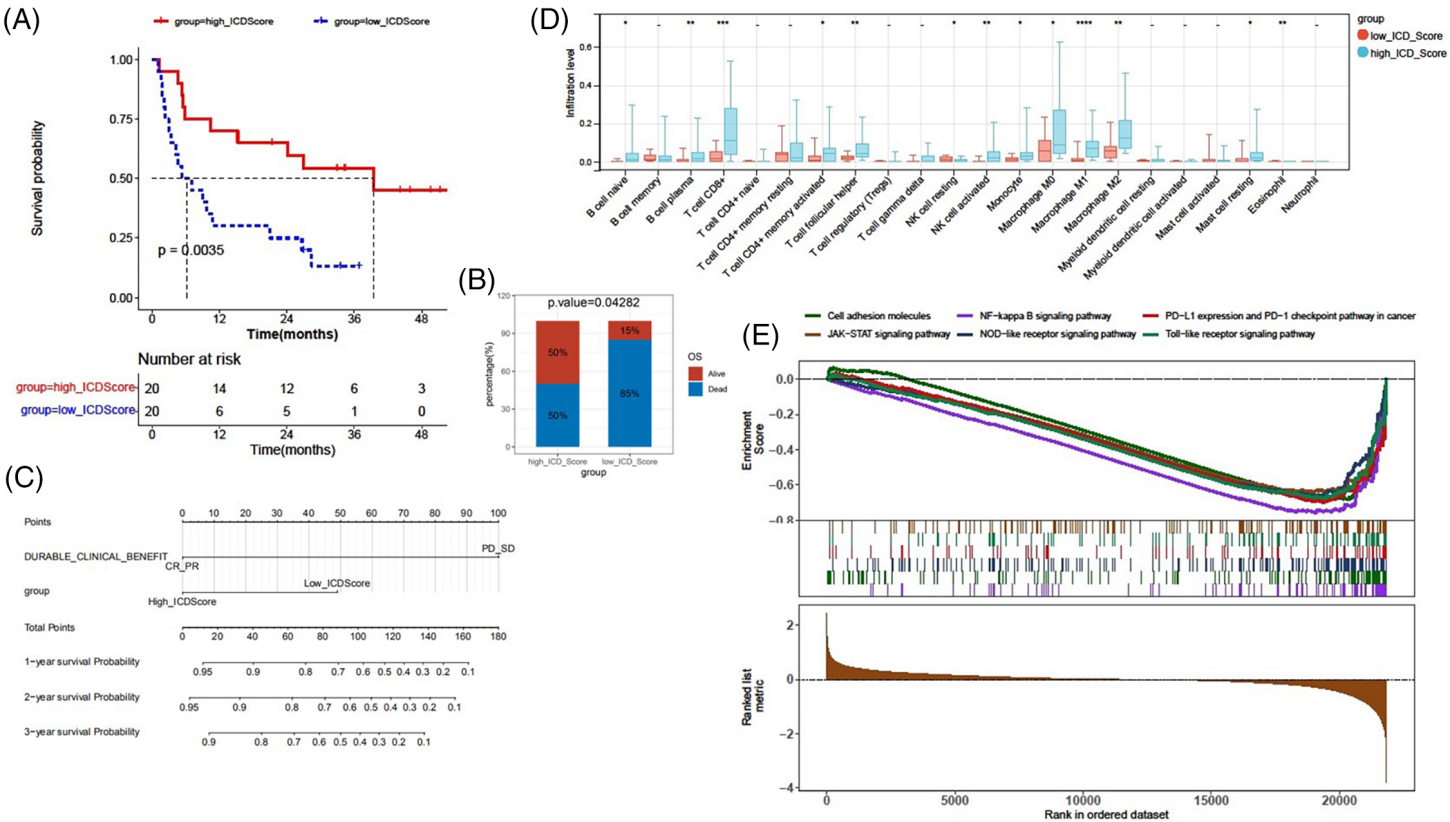
Immunogenic features between the high immunogenic cell death (ICD) cohort and the low ICD cohort in the SKCM DFCI 2015 cohort. (A) Kaplan–Meier OS curves. (B) Survival status after immunotherapy was compared using the t‐test. (C) Nomogram predicting patient survival rates. (D) Infiltration levels of 22 kinds of immune cells in tumor microenvironment grouped by ICD score were compared using the Wilcoxon test. (E) GSEA identifies variations in the enrichment hallmark pathways between the high ICD cohort and the low ICD cohort. Statistical significance was set at *p* < .05.

Tumor microenvironment plays a crucial role in dictating therapeutic outcomes, and the subtypes of tumor microenvironment can act as immunotherapy biomarkers.^24^ Could the ICD score serve as a biomarker for immunotherapy? To further validate the role of the ICD score in immunogenic features and immunotherapy response, an immunotherapy cohort, the SKCM DFCI 2015 cohort was selected for subsequent analyses. A high ICD score was found to correlate with a better survival status after immunotherapy (Figure 8A,B). Patients with high ICD scores exhibited higher complete response (CR) or partial response (PR) rates after immunotherapy, but there was no statistical difference between patients with high and low ICD scores (Figure S4A). This discrepancy might be due to the limited number of patients achieving PR or CR, and that most patients in this cohort had progressive disease (PD) or stable disease (SD). We conducted univariate and multivariate Cox regression analyses, and generated a nomogram based on the independent risk factors: durable clinical benefit (PD_SD vs. PR_CR) and ICD score (high versus low) (Table 1 and Figure 8C). The calibration curves of the nomogram‐predicted possibilities of 1‐year, 2‐year, and 3‐year OS are shown in Figure S4B. The infiltration levels of 22 types of immune cells were assessed. Patients in the high ICD cohorts exhibited noticeably elevated percentages of CD8^+^ T cells, B cell plasma, memory activated T cells, follicular helper T cells, activated NK cells, M1 macrophages, and eosinophil cells (Figure 8D). The ratios of M1 and M2 macrophage percentages were higher in the high ICD cohorts compared to the low ICD cohorts (Figure S5A). The correlations of the ICD score with CD8^+^ T cells, M1 macrophages, or activated NK cells were shown in Figure S5B. We also found strong correlations between the ICD score and the immune score, stromal score, and ESTIMATE score (Figure S5C). We found that most of the immune checkpoint genes, particularly CD27, CTLA4, LAG3, PDCD1, and PDCD1LG2, were up‐regulated in the high ICD cohorts compared to the low ICD cohorts (Figure S5D). Additionally, GSEA revealed that a high ICD score was positively associated with six hallmark pathways, namely the NF‐kappa B signaling pathway, PD‐L1 expression and PD‐L1 checkpoint pathway in cancer, JAK–STAT signaling pathway, cell adhesion molecules, NOD‐like receptor signaling pathway, and Toll‐like receptor signaling pathway (Figure 8E). Our findings indicate that the ICD score is significantly associated with the tumor microenvironment and the response to immunotherapy in the immunotherapy cohort.

## 4 DISCUSSION

ICD represents a functionally unique response pattern that involves the induction of cellular stress and culminating in cell death accompanied by the release of various DAMPs.^5^ Notably, ICD has been demonstrated to activate the tumor‐specific immune response.^2^, ^25^ Induction of ICD can be a potential treatment strategy. However, ICD biomarkers and their relationships with the tumor microenvironment, clinical features, and immunotherapy response in a clinical context are not fully understood. Therefore, the pan‐cancer analyses of ICD gene signatures obtained from a previous meta‐analysis were conducted on 33 cancers from the TCGA database. We identified several key genes, including those coding for canonical DAMPs such as CLAR and HMGB1, as well as *IFNB1*, *IFNG*, *CD8A*, *CD8B*, *CXCR3*, *FOXP3*, *BAX*, *IL6*, and *PRF1*, which were dysregulated in most cancers and possessed significant prognostic value. Moreover, we validated the correlations between the ICD genes and the tumor microenvironment in pan‐cancer and captured some key genes, such as *CD4*, *CD8A*, *CD8B*, *CXCR3*, *FOXP3*, *PRF1*, and *IFNG*, which shared strong relationships with the tumor microenvironment, immune cell infiltration, and immune checkpoints.

However, the relationship between ICD genes and the tumor microenvironment in pan‐cancer remains unclear because the whole picture cannot be seen through any single ICD gene. We calculated the ICD score, which emerged as a more comprehensive and straightforward biomarker of ICD compared to ICD genes. We identified two ICD cohorts: a high ICD score was associated with the immune‐hot phenotype, and a low ICD score was associated with the immune‐cold phenotype. Moreover, we determined the prognostic and clinical value of the ICD score in pan‐cancer and discovered the relevance between the ICD score and immunotherapy response in the SKCM DFCI 2015 cohort. A recent pan‐cancer analysis obtained results akin to ours: the ICD score could predict patient prognosis and treatment response.^26^ Our study, compared to this research, conducted a more in‐depth analysis of patient survival, immune microenvironment, and clinical features based on the subtypes of the ICD score. In previous studies, the high‐ICD subtype, derived from ICD genes, has been considered a protective factor for prognosis in HNSCC, lung adenocarcinoma, and ESCA, but a risk factor in pancreatic cancer and glioblastoma multiforme.^10^, ^11^, ^12^, ^13^, ^27^ However, our results showed that a high ICD score was a protective factor for ACC, SKCM, and SARC, but a risk factor for UVM and LAML. The reason for differences in analysis results may be the method of consensus clustering used to distinguish ICD‐associated subtypes. In addition, we confirmed that radiotherapy could elevate patient ICD scores in some cancer types. This indicates that radiotherapy may shift the patient's immune phenotype from “cold” to “hot”, with the underlying mechanism involving the regulation of immune and inflammation pathways.

While immunotherapy has revolutionized cancer therapy, monotherapy is often insufficient for many patients. The combination of ICD inducers, such as certain chemotherapeutic drugs, radiotherapy, and photodynamic therapy, with immunotherapy, provides great therapeutic advantages.^2^ To discover more ICD inducers, we aimed to predict therapeutic agents that target ICD genes. It was validated that not only generally recognized ICD inducers, such as bleomycin, doxorubicin, bortezomib and cyclophosphamide,^5^ but also gemcitabine was found to target a majority of the ICD genes. The combination of gemcitabine‐containing chemotherapy with ICI therapy has demonstrated encouraging outcomes in the clinical treatment of several cancers, including pancreatic cancer, cholangiocarcinoma bladder cancer and HNSCC.^17^, ^28^, ^29^ Many patients exhibit limited sensitivity to ICI monotherapy, making the combination therapy necessary. Chen et al. reported that gemcitabine‐containing chemoimmunotherapy improved the response rate of stage II‐III muscle invasive bladder cancer compared to ICI monotherapy.^17^ Chemotherapy modulates tumor immunity in a drug‐dependent manner. It was demonstrated that patients with non‐small cell lung cancer who received pre‐ICI gemcitabine‐containing chemotherapy had longer progression free survival and a better response to ICI therapy than those who received other chemotherapeutic regimens.^24^ The immunomodulatory effects of gemcitabine include promoting the expression of immunogenic molecules on of cancer cells, releasing immunogenic substances, and altering the tumor microenvironment.^18^, ^30^, ^31^, ^32^ In our study, we also analyzed the potential mechanisms of gemcitabine targeting ICD genes by performing GO and KEGG enrichment analyses. We found that gemcitabine‐targeting genes were particularly enriched in the PD‐L1 expression and PD1 checkpoint pathway in cancer. Our results suggest that gemcitabine may be considered an ICD inducer by converting “cold” cancer to “hot” cancer. However, a previous study suggested that gemcitabine was insufficient to induce ICD in bladder cancer, despite it potentiating the release of hallmark DAMPs from dying cancer cells.^33^ Therefore, more research is needed to determine whether gemcitabine has an ICD‐inducing effect in various cancers.

In conclusion, we have conducted the first comprehensive analyses of the ICD gene signatures across 33 distinct cancer types. Our study emphasizes the correlations between the ICD score and patient prognosis, clinical features, the immunological tumor microenvironment, and the therapeutic response to immunotherapy. These findings indicate that the ICD gene signatures could potentially serve as biomarkers for ICD. Our research provides novel insights into immunophenotypic assessment and cancer therapeutic strategies, which could help to broaden the application of immunotherapy to benefit more patients.

## AUTHOR CONTRIBUTIONS

**Xiaodan Han:** Formal analysis (equal); funding acquisition (lead); visualization (lead); writing – original draft (equal). **Di Song:** Formal analysis (equal); software (equal); writing – original draft (equal). **Yongliang Cui:** Formal analysis (equal); software (equal); writing – review and editing (equal). **Yonggang Shi:** Resources (lead); writing – review and editing (equal). **Xiaobin Gu:** Conceptualization (equal); supervision (equal); writing – review and editing (lead).

## CONFLICT OF INTEREST STATEMENT

The authors have stated explicitly that there are no conflicts of interest in connection with this article.

## ETHICS STATEMENT

Ethical is not applicable because these data are from public database.

## Supporting information


**Appendix S1:** Supporting Information.

## Data Availability

The original contributions presented in the study are publicly available. The data can be found in the TCGA database (https://portal.gdc.cancer.gov/). Further inquiries can be directed to the corresponding author.

## References

[1] Galluzzi L , Vitale I , Warren S , et al. Consensus guidelines for the definition, detection and interpretation of immunogenic cell death. J Immunother Cancer. 2020;8:8. 10.1136/jitc-2019-000337 PMC7064135 32209603

[2] Ahmed A , Tait SWG . Targeting immunogenic cell death in cancer. Mol Oncol. 2020;14:2994‐3006. 33179413 10.1002/1878-0261.12851 PMC7718954

[3] Galluzzi L , Buque A , Kepp O , et al. Immunogenic cell death in cancer and infectious disease. Nat Rev Immunol. 2017;17:97‐111. 27748397 10.1038/nri.2016.107

[4] Fabian KP , Wolfson B , Hodge JW . From immunogenic cell death to immunogenic modulation: select chemotherapy regimens induce a Spectrum of immune‐enhancing activities in the tumor microenvironment. Front Oncol. 2021;11:728018. 34497771 10.3389/fonc.2021.728018 PMC8419351

[5] Fucikova J , Kepp O , Kasikova L , et al. Detection of immunogenic cell death and its relevance for cancer therapy. Cell Death Dis. 2020;11:1013. 33243969 10.1038/s41419-020-03221-2 PMC7691519

[6] Fucikova J , Moserova I , Urbanova L , et al. Prognostic and predictive value of DAMPs and DAMP‐associated processes in cancer. Front Immunol. 2015;6:402. 26300886 10.3389/fimmu.2015.00402 PMC4528281

[7] Humeau J , Bezu L , Kepp O , Kroemer G . EIF2alpha phosphorylation: a hallmark of both autophagy and immunogenic cell death. Mol Cell Oncol. 2020;7:1776570. 32944635 10.1080/23723556.2020.1776570 PMC7469655

[8] Krysko DV , Garg AD , Kaczmarek A , Krysko O , Agostinis P , Vandenabeele P . Immunogenic cell death and DAMPs in cancer therapy. Nat Rev Cancer. 2012;12:860‐875. 23151605 10.1038/nrc3380

[9] Garg AD , De Ruysscher D , Agostinis P . Immunological metagene signatures derived from immunogenic cancer cell death associate with improved survival of patients with lung, breast or ovarian malignancies: a large‐scale meta‐analysis. Onco Targets Ther. 2016;5:e1069938. 10.1080/2162402X.2015.1069938 PMC4801472 27057433

[10] Wang X , Wu S , Liu F , et al. An immunogenic cell death‐related classification predicts prognosis and response to immunotherapy in head and neck squamous cell carcinoma. Front Immunol. 2021;12:781466. 34868055 10.3389/fimmu.2021.781466 PMC8640500

[11] Cui Y , Li Y , Long S , et al. Comprehensive analysis of the immunogenic cell death‐related signature for predicting prognosis and immunotherapy efficiency in patients with lung adenocarcinoma. BMC Med Genet. 2023;16(1):184. 10.1186/s12920-023-01604-w PMC10410984 37553698

[12] Liu Z , Li W , You G , Hu Z , Liu Y , Zheng N . Genomic analysis of immunogenic cell death‐related subtypes for predicting prognosis and immunotherapy outcomes in glioblastoma multiforme. Open Med. 2023;18(1):20230716. 10.1515/med-2023-0716 PMC10238813 37273917

[13] Yu W , Li M , Xia J . Identification of immunogenic cell death‐related prognostic signatures in pancreatic cancer. Oncol Lett. 2023;26(5):473. 37809045 10.3892/ol.2023.14061 PMC10551861

[14] Van Allen EM , Miao D , Schilling B , et al. Genomic correlates of response to CTLA‐4 blockade in metastatic melanoma. Science. 2015;350(6257):207‐211. 26359337 10.1126/science.aad0095 PMC5054517

[15] Geeleher P , Cox N , Huang RS . pRRophetic: an R package for prediction of clinical chemotherapeutic response from tumor gene expression levels. PLoS One. 2014;9:e107468. 25229481 10.1371/journal.pone.0107468 PMC4167990

[16] Geeleher P , Cox NJ , Huang RS . Clinical drug response can be predicted using baseline gene expression levels and in vitro drug sensitivity in cell lines. Genome Biol. 2014;15:R47. 24580837 10.1186/gb-2014-15-3-r47 PMC4054092

[17] Arico E , Castiello L , Capone I , et al. Type I interferons and cancer: an evolving story demanding novel clinical applications. Cancers. 2019;11:11. 10.3390/cancers11121943 PMC6966569 31817234

[18] Koido S , Kan S , Yoshida K , et al. Immunogenic modulation of cholangiocarcinoma cells by chemoimmunotherapy. Anticancer Res. 2014;34:6353‐6361. 25368235

[19] Suzuki E , Kapoor V , Jassar AS , Kaiser LR , Albelda SM . Gemcitabine selectively eliminates splenic gr‐1+/CD11b+ myeloid suppressor cells in tumor‐bearing animals and enhances antitumor immune activity. Clin Cancer Res. 2005;11:6713‐6721. 16166452 10.1158/1078-0432.CCR-05-0883

[20] Principe DR , Narbutis M , Kumar S , et al. Long‐term gemcitabine treatment reshapes the pancreatic tumor microenvironment and sensitizes murine carcinoma to combination immunotherapy. Cancer Res. 2020;80:3101‐3115. 32238357 10.1158/0008-5472.CAN-19-2959 PMC7777391

[21] Alonso MH , Ausso S , Lopez‐Doriga A , et al. Comprehensive analysis of copy number aberrations in microsatellite stable colon cancer in view of stromal component. Br J Cancer. 2017;117:421‐431. 28683472 10.1038/bjc.2017.208 PMC5537504

[22] Gong X , Karchin R . Pan‐cancer HLA gene‐mediated tumor immunogenicity and immune evasion. Mol Cancer Res. 2022;20(8):1272‐1283. 35533264 10.1158/1541-7786.MCR-21-0886 PMC9357147

[23] Chowell D , Morris LGT , Grigg CM , et al. Patient HLA class I genotype influences cancer response to checkpoint blockade immunotherapy. Science. 2018;359(6375):582‐587. 29217585 10.1126/science.aao4572 PMC6057471

[24] Bagaev A , Kotlov N , Nomie K , et al. Conserved pan‐cancer microenvironment subtypes predict response to immunotherapy. Cancer Cell. 2021;39(6):845‐865. 34019806 10.1016/j.ccell.2021.04.014

[25] Wang Q , Ju X , Wang J , Fan Y , Ren M , Zhang H . Immunogenic cell death in anticancer chemotherapy and its impact on clinical studies. Cancer Lett. 2018;438:17‐23. 30217563 10.1016/j.canlet.2018.08.028

[26] Wang Y , Huang Y , Yang M , et al. Comprehensive Pan‐cancer analyses of immunogenic cell death as a biomarker in predicting prognosis and therapeutic response. Cancers. 2022;14(23):5952. 36497433 10.3390/cancers14235952 PMC9736000

[27] Zhang Y , Chen Y . Stratification from heterogeneity of the cell‐death signal enables prognosis prediction and immune microenvironment characterization in esophageal squamous cell carcinoma. Front Cell Dev Biol. 2022;10:855404. 35493093 10.3389/fcell.2022.855404 PMC9040162

[28] Gao G , Jia K , Zhao S , et al. Analysis of the association between prior chemotherapy regimens and outcomes of subsequent anti‐PD‐(L)1 monotherapy in advanced non‐small cell lung cancer. Transl Lung Cancer Res. 2019;8:920‐928. 32010570 10.21037/tlcr.2019.11.25 PMC6976343

[29] Shui L , Cheng K , Li X , et al. Durable response and good tolerance to the triple combination of Toripalimab, gemcitabine, and nab‐paclitaxel in a patient with metastatic pancreatic ductal adenocarcinoma. Front Immunol. 2020;11:1127. 32636837 10.3389/fimmu.2020.01127 PMC7318868

[30] Liu WM , Fowler DW , Smith P , Dalgleish AG . Pre‐treatment with chemotherapy can enhance the antigenicity and immunogenicity of tumours by promoting adaptive immune responses. Br J Cancer. 2010;102:115‐123. 19997099 10.1038/sj.bjc.6605465 PMC2813751

[31] Dijkgraaf EM , Santegoets SJ , Reyners AK , et al. A phase 1/2 study combining gemcitabine, Pegintron and p53 SLP vaccine in patients with platinum‐resistant ovarian cancer. Oncotarget. 2015;6:32228‐32243. 26334096 10.18632/oncotarget.4772 PMC4741673

[32] Pei Q , Pan J , Zhu H , et al. Gemcitabine‐treated pancreatic cancer cell medium induces the specific CTL antitumor activity by stimulating the maturation of dendritic cells. Int Immunopharmacol. 2014;19:10‐16. 24389382 10.1016/j.intimp.2013.12.022

[33] Hayashi K , Nikolos F , Lee YC , et al. Tipping the immunostimulatory and inhibitory DAMP balance to harness immunogenic cell death. Nat Commun. 2020;11:6299. 33288764 10.1038/s41467-020-19970-9 PMC7721802

