# Supplementary material for: Pan‐cancer analyses of immunogenic cell death‐derived gene signatures: Potential biomarkers for prognosis and immunotherapy
Source: Cancer Rep (Hoboken). 2024 Apr 16;7(4):e2073. doi: 10.1002/cnr2.2073 (PMC11021686; doi:10.1002/cnr2.2073)
Supplement: Supplementary file 1 — Appendix S1: Supporting Information. [file CNR2-7-e2073-s001.docx]

Pan-cancer Analyses of Immunogenic Cell Death-derived Gene Signatures: Potential Biomarkers for Prognosis and Immunotherapy


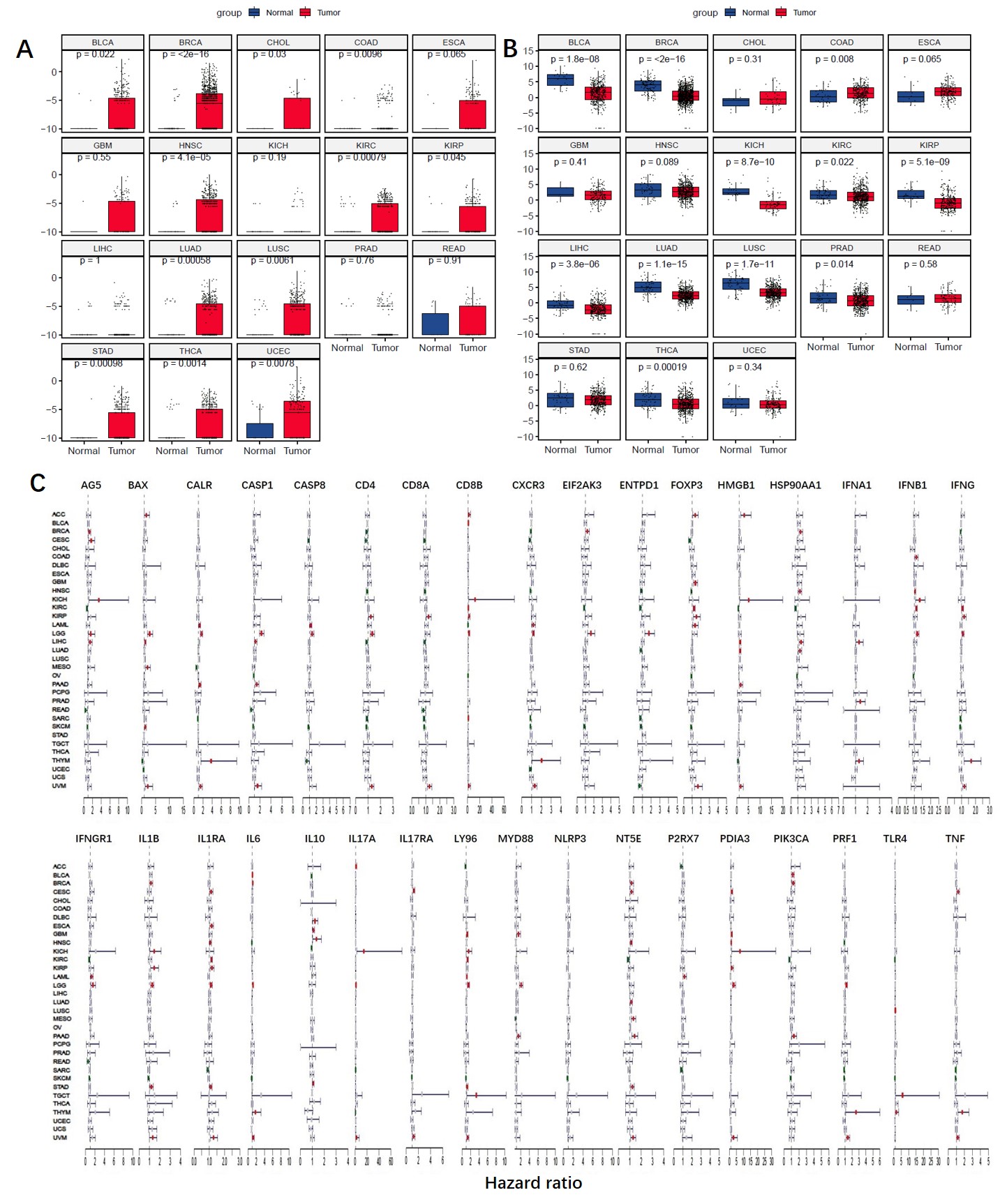


Figure S1. Expression and univariate Cox analysis of ICD genes in pan-cancer. (A) Expression of IFNB1 in tumor and adjacent normal samples was compared using the Wilcoxon test. (B) Expression of IL6 in tumor and adjacent normal samples was compared using the Wilcoxon test. (C) Univariate Cox analysis evaluating the prognostic significance of the ICD genes in terms of OS. Forest plots of the hazard risk (HR) with 95% confidence intervals showing an advantage (HR<1, green) or a disadvantage (HR>1, red) in the OS with increasing ICD gene expression. Statistical significance was set at p < 0.05.


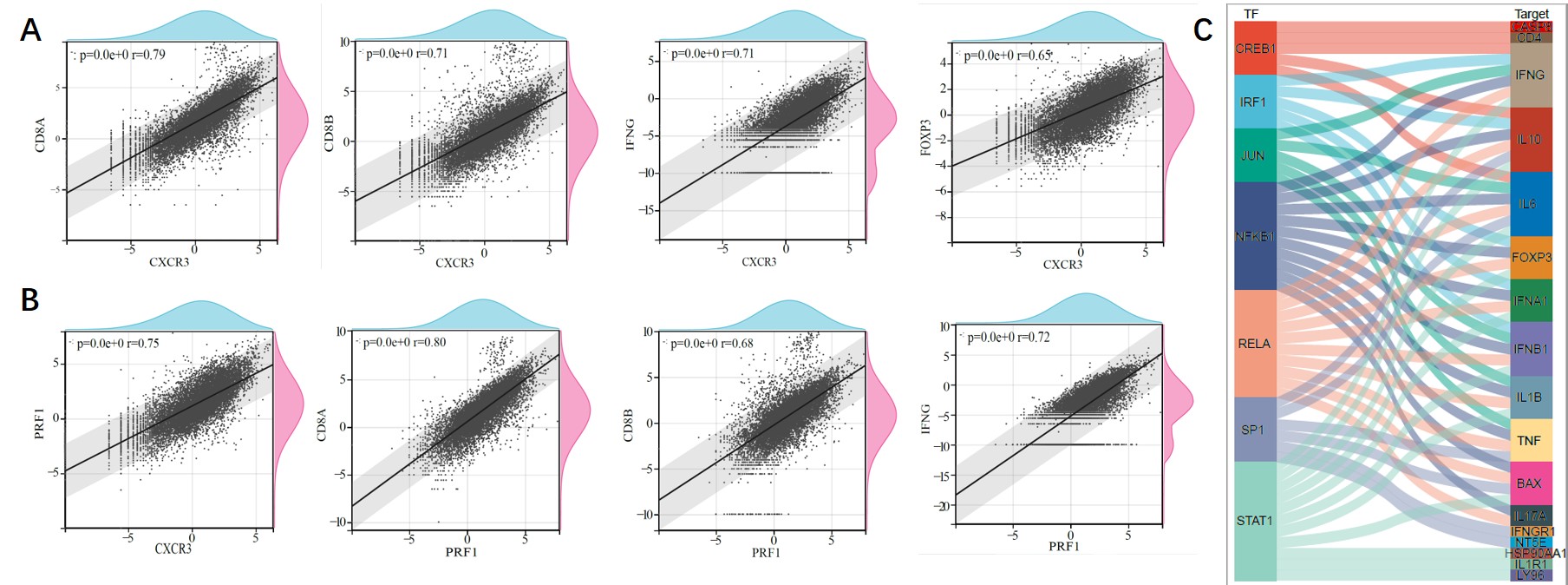


Figure S2. Identification of correlations among ICD molecules and prediction of TFs. (A) Correlations between CXCR3 and other ICD gene expressions. (B) Correlations between PRF1 and other ICD gene expressions. The Pearson's Correlation test was used for p-value calculation. (C) Sankey diagram showing associations between ICD-related TFs and ICD genes. Statistical significance was set at p < 0.05.


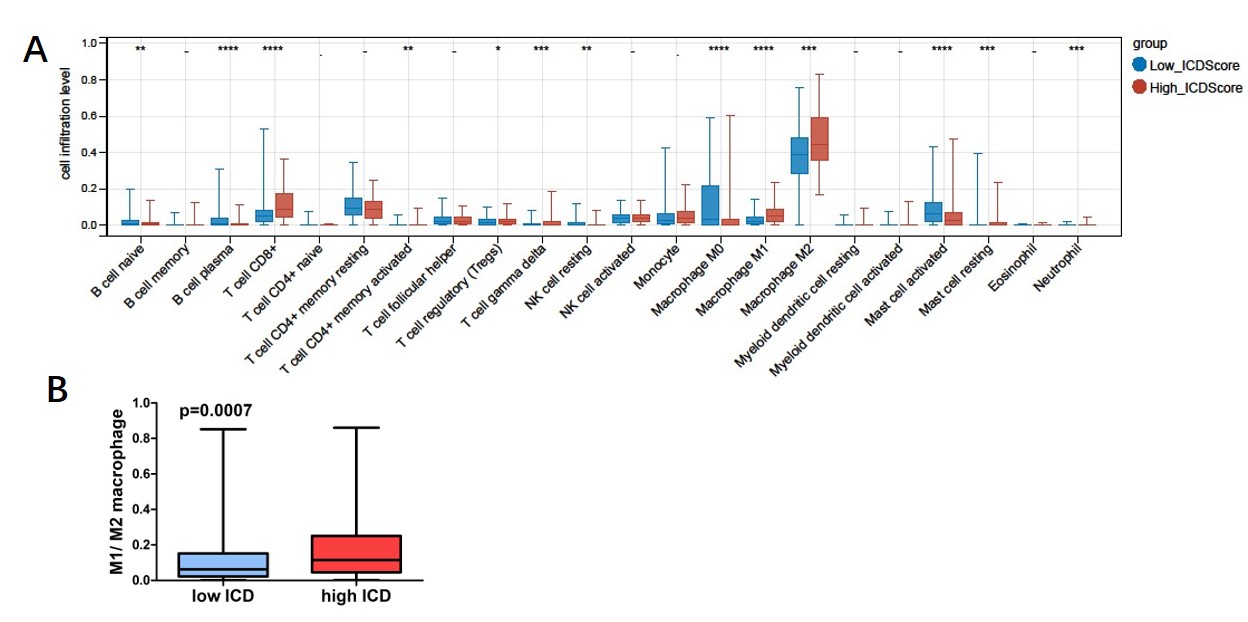


Figure S3. Infiltration levels of immune cells in high ICD cohorts and low ICD cohorts in SARC. (A) Infiltration levels of 22 kinds of immune cells in the tumor microenvironment grouped by ICD score were compared using the Wilcoxon test. (B) The ratio of M1 macrophage and M2 macrophage in the tumor microenvironment grouped by ICD score were compared using the t-test. Statistical significance was set at p < 0.05.


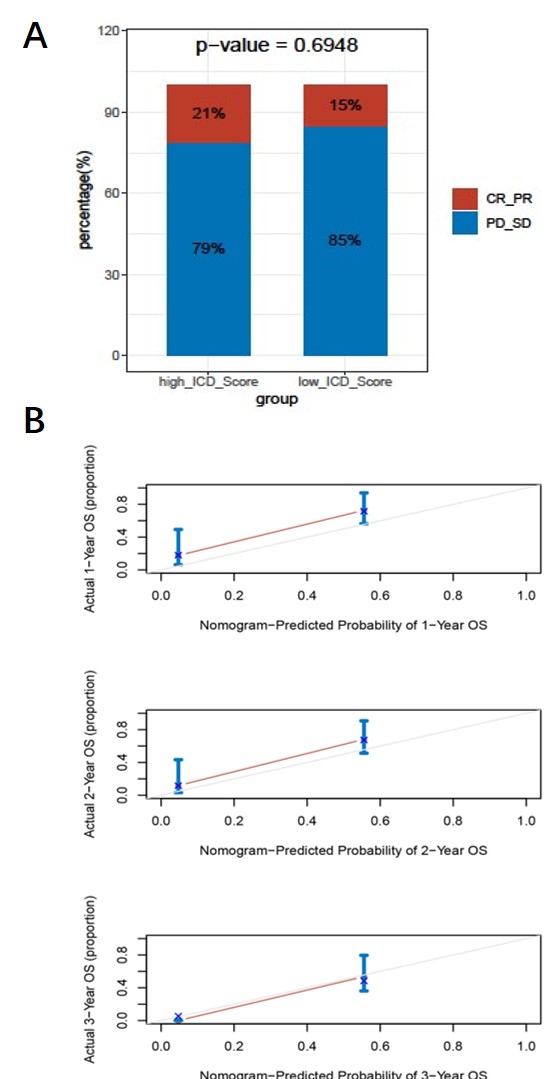


Figure S4. Patient response rate and calibration curves of the nomogram for patient survival prediction after immunotherapy in SKCM DFCI 2015 cohort. (A) The patient response (CR: complete response; PR: partial response; PD: progressive disease; SD: stable disease) rate grouped by ICD score were compared using the t-test. (B) Calibration curves of the nomogram-predicted possibilities of 1-year (up), 2-year (middle) and 3-year (down) OS after immunotherapy.


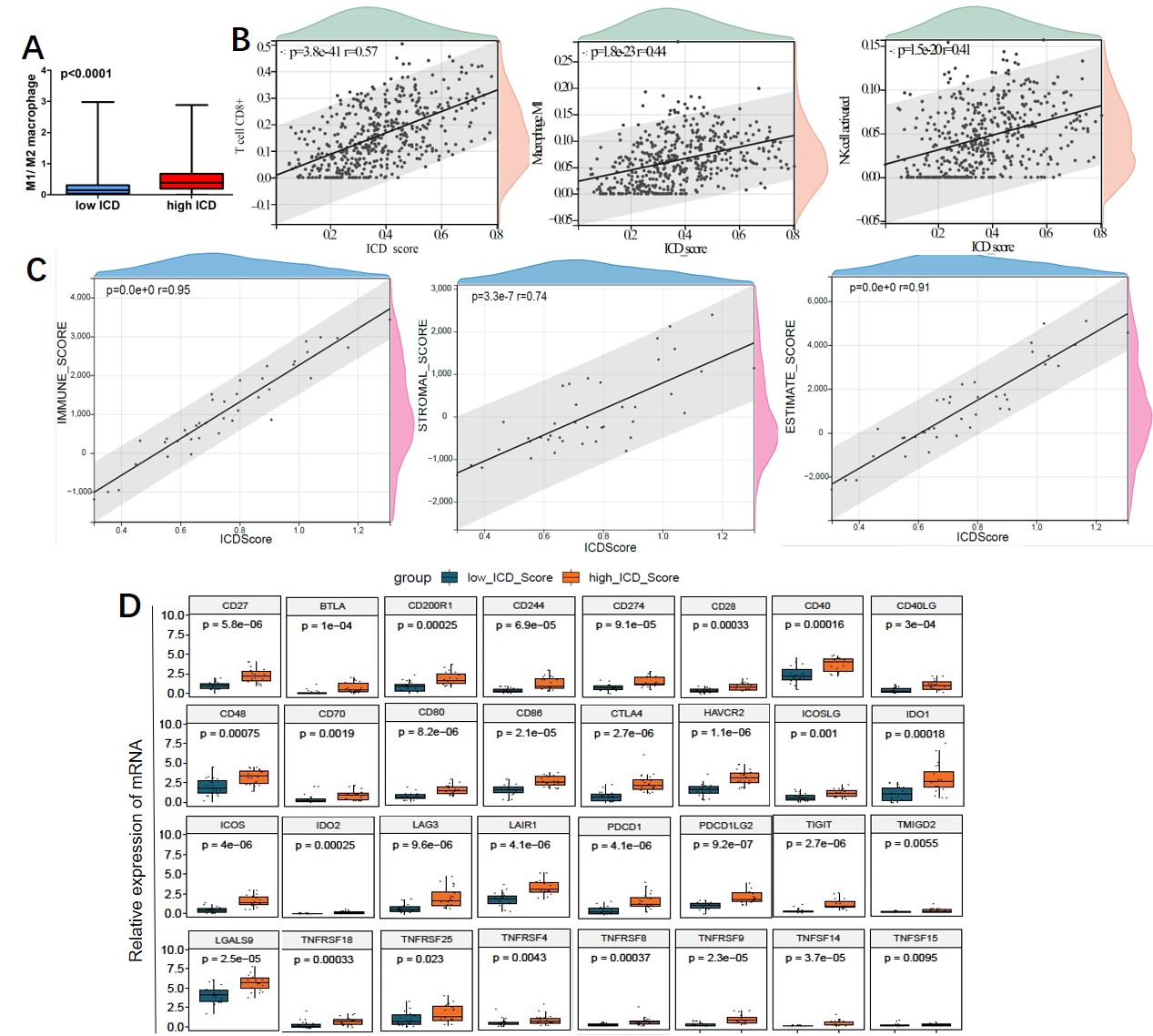


Figure S5. Correlation analysis between the ICD score and the tumor microenvironment in SKCM DFCI 2015 cohort. (A) The ratio of M1 macrophage and M2 macrophage in the tumor microenvironment in high ICD cohorts and low ICD cohorts were compared using the t-test. (B) Correlations between CD8^+^ T cells, M1 macrophages, activated NK cells versus the ICD score. (C) Correlations between the immune score, stromal score, ESTIMATE score versus the ICD score. The Pearson's Correlation test was used for p-value calculation. (D) Relative expression levels of some immune checkpoint genes in high ICD cohorts and low ICD cohorts were compared using the Wilcoxon test.

Table 1. Univariate and multivariate Cox regression analyses in SKCM DFCI 2015 cohort.

|  | Univariate Cox regression | | | | Multivariate Cox regression | | | |
| --- | --- | --- | --- | --- | --- | --- | --- | --- |
|  | HR | lower.95 | upper.95 | p.val | HR | lower.95 | upper.95 | p.val |
| Sex(male/female) | 0.511 | 0.238 | 1.096 | 0.085 |  |  |  |  |
| Age(>60/<=60) | 0.776 | 0.364 | 1.657 | 0.513 |  |  |  |  |
| Durable clinical benefit (PD_SD/PR_CR) | 10.707 | 1.445 | 79.314 | 0.020 | 12.391 | 1.660 | 92.513 | 0.014 |
| ICD_score(High/Low) | 0.315 | 0.139 | 0.713 | 0.006 | 0.292 | 0.127 | 0.673 | 0.004 |
